# Tabernanthalog, a Non-Hallucinogenic Psychedelic, Alleviates Cancer-Induced Cognitive Deficits via Serotonergic Pathways

**DOI:** 10.3390/ijms26157519

**Published:** 2025-08-04

**Authors:** Masahide Arinaga, Jun Yamada, Shoichiro Maeda, Ayumi Okamura, Yuto Oshima, Liye Zhang, Yiying Han, Kyoko M. Iinuma, Shozo Jinno

**Affiliations:** Department of Anatomy and Neuroscience, Graduate School of Medical Sciences, Kyushu University, 3-1-1 Maidashi, Higashi-ku, Fukuoka 812-8582, Japan; arinaga.masahide.908@s.kyushu-u.ac.jp (M.A.);

**Keywords:** cancer, cognitive impairment, serotonin, neuroinflammation, tabernanthalog

## Abstract

Cancer-related cognitive impairment (CRCI)—encompassing anxiety, depression, and memory deficits—significantly diminishes the quality of life in patients with cancer, yet remains underrecognized in clinical practice. In this study, we investigated the therapeutic potential of tabernanthalog (TBG), a non-hallucinogenic analog of psychedelic compounds, as a novel intervention for CRCI using a Lewis lung carcinoma (3LL) mouse model. Behavioral assessments revealed heightened anxiety-like behavior and memory impairment following 3LL cell transplantation. Biochemical analysis revealed reduced tryptophan levels in both blood and hippocampal tissue, accompanied by the downregulation of serotonergic receptor genes and upregulation of pro-inflammatory cytokine genes in the hippocampus of tumor-bearing mice. Additionally, microglial density and morphological activation were markedly elevated. TBG treatment reversed these behavioral deficits, improving both anxiety-related behavior and memory performance. These effects were associated with the normalization of microglial density and morphology, as well as the restoration of serotonergic receptor and cytokine gene expression. In vitro, TBG partially suppressed neuroinflammatory gene expression in BV-2 microglial cells exposed to conditioned medium from 3LL cells. Collectively, these findings suggest that TBG alleviates CRCI-like symptoms by modulating neuroinflammation and microglial activation. This study highlights TBG as a promising therapeutic candidate for improving cognitive and emotional functioning in patients with cancer.

## 1. Introduction

Cancer-related cognitive impairment (CRCI) presents a substantial clinical challenge, affecting a large proportion of individuals diagnosed with cancer [1]. Far from a minor side effect, CRCI encompasses persistent and often debilitating cognitive deficits spanning multiple domains, including memory, attention, executive function, and mood regulation. The interplay between cancer, its treatments, and brain function is complex and multifaceted [2]. Among the proposed mechanisms, neuroinflammation has emerged as a key etiological factor in CRCI [3]. Cancer-associated cytokines and chemokines may cross a compromised blood–brain barrier (BBB), initiating inflammatory cascades and disrupting neural function [4]. The BBB, a selective barrier that normally protects the brain from harmful substances in the bloodstream, can be impaired by both tumors and their treatment. Additionally, cancer can alter neurotransmitter homeostasis, further contributing to cognitive and emotional disturbances [5].

Depression severely impairs the quality of life of patients with cancer, exacerbating the overall burden of the disease. However, the efficacy of conventional antidepressants, such as selective serotonin reuptake inhibitors (SSRIs) and serotonin–norepinephrine reuptake inhibitors (SNRIs), remains uncertain in this population [6]. Notably, cancer alters amino acid metabolism, particularly that of tryptophan, which is a precursor of serotonin, a neurotransmitter crucial for the regulation of mood, sleep, and cognition [7]. Cancer cells often exhibit increased tryptophan catabolism through the activation of the enzyme indoleamine 2,3-dioxygenase (IDO). This metabolic shift depletes circulating tryptophan levels in patients with lung cancer [8]. Decreased tryptophan availability may be associated with reduced serotonin concentrations and, consequently, fatigue and depression in patients with cancer [9].

Emerging evidence underscores the critical role of serotonin in modulating neuroinflammatory responses, suggesting a bidirectional relationship between serotonergic signaling and immune activation [10]. Pro-inflammatory cytokines, such as tumor necrosis factor-alpha (TNF-α) and interleukin-6 (IL-6), can activate IDO, enhancing tryptophan catabolism and subsequently diminishing serotonin synthesis [11]. Conversely, serotonin modulates immune responses via its receptors (5-hydroxytrptamine, 5-HT) expressed on immune cells [12]. Microglia, the primary immune cells of the central nervous system, maintain neural homeostasis through the continuous surveillance of their microenvironment [13]. However, chronic psychological stress can activate microglia, leading to the release of pro-inflammatory cytokines, a mechanism implicated in the pathogenesis of depression [14]. Notably, antidepressants, including SSRIs and SNRIs, prevent microglial activation in animal models of neuroinflammation [15].

Recent research indicates that classic hallucinogenic psychedelics, including psilocybin, lysergic acid diethylamide (LSD), and dimethyltryptamine (DMT), which are structurally diverse serotonin 5-HT_2A_ receptor agonists, may offer substantial therapeutic benefits for depression, particularly when conventional antidepressant treatments are unsuccessful [16]. For instance, psilocybin has shown promise in alleviating depression and anxiety in patients with terminal illnesses [17], while LSD-assisted therapy has demonstrated sustained anxiolytic effects [18]. Tabernanthalog (TBG), a water-soluble, non-hallucinogenic, and non-toxic analog of ibogaine, has been observed to elicit antidepressant-like effects via the activation of 5-HT_2A_ receptors [19].

In this study, we used a murine model of CRCI by transplanting Lewis lung carcinoma (3LL) cells into the dorsal flank of mice. We aimed to elucidate the potential effects of TBG on cognitive and emotional behaviors, hippocampal gene expression profiles, and microglial activity in tumor-bearing mice.

## 2. Results

### 2.1. Lung Cancer Induces Anxiety-like Behavior and Contextual Memory Impairment in Mice

**Figure 1:**
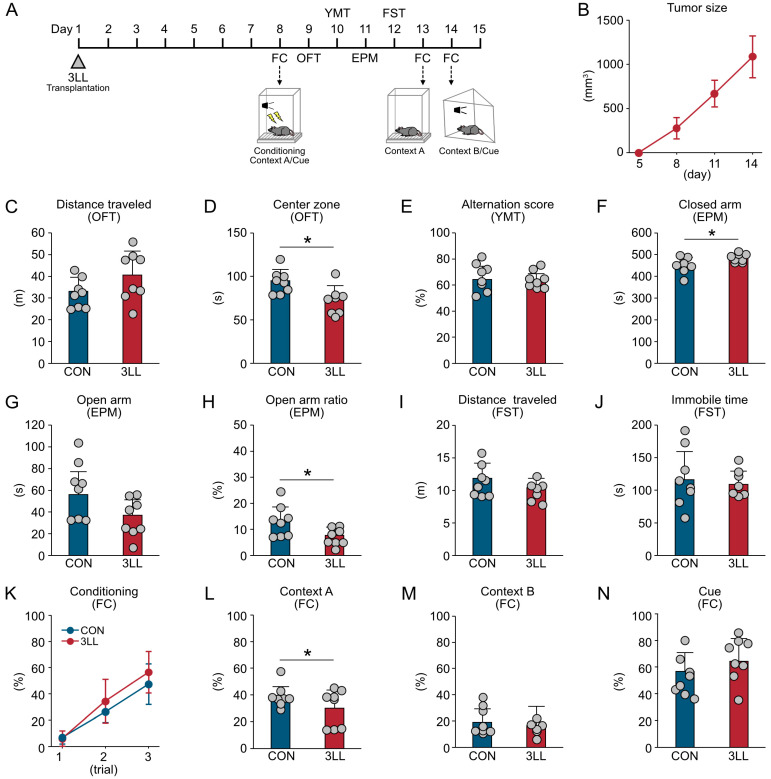
Lung cancer transplantation induces anxiety-like behavior and memory impairment in mice. (**A**) Schematic timeline depicting the transplantation of 3LL Lewis lung carcinoma cells and the subsequent behavioral test battery schedule, including the OFT, YMT, EPM test, FST, and FC test. (**B**) Tumor growth progression in 3LL mice. (**C**,**D**) Total distance traveled (m) (**C**) and time spent in the center zone (s) (**D**) during the OFT. (**E**) Spontaneous alternation score (%) in the YMT. (**F**–**H**) Time spent in the closed arms (s) (**F**), open arms (s) (**G**), and open arm entry ratio (%) (**H**) in the EPM. (**I**,**J**) Distance traveled (m) (**I**) and immobility time (s) (**J**) in the FST. (**K**) Freezing time ratio (%) during the conditioning phase in context A. (**L**,**M**) Freezing time ratio (%) during memory recall in contexts A (**L**) and B (**M**). (**N**) Freezing time ratio (%) in response to the auditory cue (tone paired with foot shock). Statistical differences were analyzed using Welch’s *t*-test. Data are presented as mean ± SD (CON, *n* = 8 mice; 3LL, *n* = 8 mice). Each grey circle represents an individual animal. Asterisks denote statistical significance: * *p* < 0.05.

To investigate the effects of lung cancer on cognitive and emotional behaviors, we assessed 3LL tumor-bearing mice (3LL mice) with control mice (CON mice) using a comprehensive behavioral test battery (Figure 1). This included the fear conditioning (FC) test, open field test (OFT), Y-maze test (YMT), elevated plus maze (EPM) test, and forced swim test (FST) (Figure 1A). As expected, tumor size progressively increased following 3LL cell transplantation (Figure 1B).

In the OFT, the total distance traveled did not differ between 3LL and CON mice, indicating intact general locomotor activity. However, 3LL mice spent less time in the center zone, suggesting heightened anxiety-like behavior (Figure 1C,D). The YMT showed no significant differences in spontaneous alternation scores between groups, indicating intact working memory in 3LL mice (Figure 1E). Consistent with the OFT findings, the EPM test further confirmed anxiety-like behavior in 3LL mice. These mice spent more time in the closed arms and exhibited a reduced open arm ratio, although the time spent in the open arms was not significantly different between groups (Figure 1F–H). In the FST, no significant differences were observed in total distance traveled or immobility time, suggesting that depression-like behavior was not markedly affected in 3LL mice (Figure 1I,J). In the FC test, freezing behavior during the conditioning phase was comparable between groups, indicating similar baseline learning (Figure 1K). However, 3LL mice exhibited a significantly reduced freezing response in context A, reflecting impaired hippocampus-dependent contextual fear memory (Figure 1L). In contrast, freezing responses in context B and to the auditory cue did not differ significantly between groups, suggesting that cued fear memory remained intact (Figure 1M,N). Collectively, these results demonstrate that lung cancer induces anxiety-like behaviors and selective contextual memory impairments in mice, without affecting general locomotor activity, working memory, or depression-like behavior.

### 2.2. Reduced Hippocampal Tryptophan Availability and Serotonin Synthesis Gene Expression in 3LL Mice

**Figure 2:**
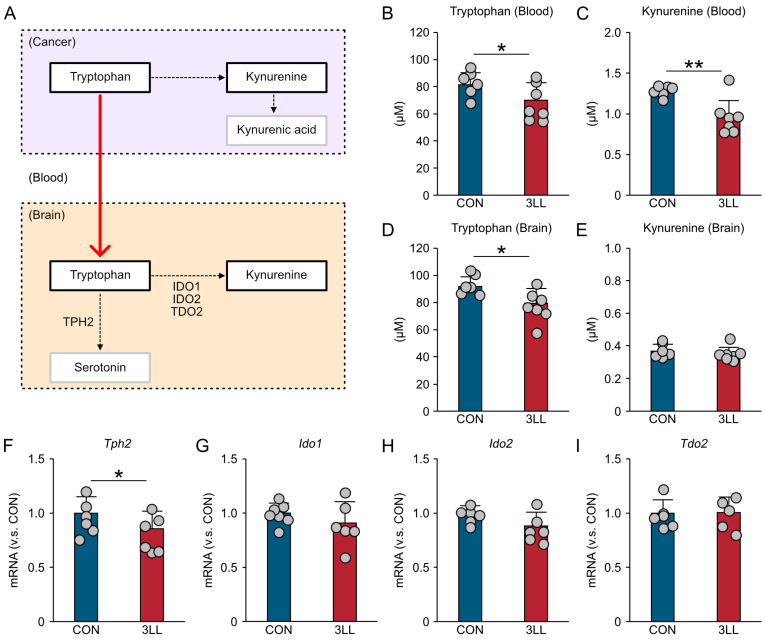
Disruptions to tryptophan metabolism in the blood and hippocampus of 3LL mice. (**A**) Schematic overview of tryptophan metabolism. (**B**,**C**) ELISA quantification of tryptophan (**B**) and kynurenine (**C**) levels in blood. (**D**,**E**) ELISA quantification of tryptophan (**D**) and kynurenine (**E**) levels in hippocampal tissue. (**F**–**I**) Relative mRNA expression levels (normalized to CON) of genes involved in serotonin synthesis and the kynurenine pathway in the hippocampus: *Tph2* (**F**), *Ido1* (**G**), *Ido2* (**H**), and *Tdo2* (**I**). Statistical differences were analyzed using Welch’s *t*-test. Data are presented as mean ± SD: tryptophan and kynurenine measurements (CON, *n* = 6 mice; 3LL, *n* = 7 mice); RT-qPCR (CON, *n* = 6 mice; 3LL, *n* = 6 mice). Each grey circle represents an individual animal. Asterisks denote statistical significance: * *p* < 0.05, ** *p* < 0.01.

Previous studies have reported abnormal tryptophan metabolism in lung cancer [8]. As tryptophan is the primary precursor for serotonin, alterations in systemic tryptophan levels may significantly impact brain serotonergic function [20]. To explore this relationship, we quantified tryptophan and kynurenine concentrations in the blood and hippocampus and assessed the hippocampal expression of key genes involved in tryptophan metabolism (Figure 2A).

An enzyme-linked immunosorbent assay (ELISA) revealed a significant reduction in blood tryptophan levels in 3LL mice compared to controls (Figure 2B). Similarly, blood kynurenine concentrations were also decreased in 3LL mice (Figure 2C). In the hippocampus, tryptophan levels were likewise reduced in 3LL mice relative to controls (Figure 2D), whereas hippocampal kynurenine levels did not differ significantly between the groups (Figure 2E).

To further investigate the molecular basis of these changes, we assessed the expression of key genes involved in tryptophan metabolism using reverse transcription quantitative polymerase chain reaction (RT-qPCR). The expression of the gene encoding tryptophan hydroxylase-2 (*Tph2*), a rate-limiting enzyme in serotonin synthesis, was significantly downregulated in the hippocampus of 3LL mice (Figure 2F). In contrast, the expression levels of kynurenine pathway genes—indoleamine 2,3-dioxygenase 1 (*Ido1*), indoleamine 2,3-dioxygenase 2 (*Ido2*), and tryptophan 2,3-dioxygenase 2 (*Tdo2*)—remained unchanged (Figure 2G–I). These findings suggest that lung cancer progression disrupts both systemic and hippocampal tryptophan homeostasis, characterized by reduced tryptophan availability and impaired serotonin synthesis in the brain.

### 2.3. Downregulation of Serotonergic Receptors and Heightened Neuroinflammation in the Hippocampus of 3LL Mice

**Figure 3:**
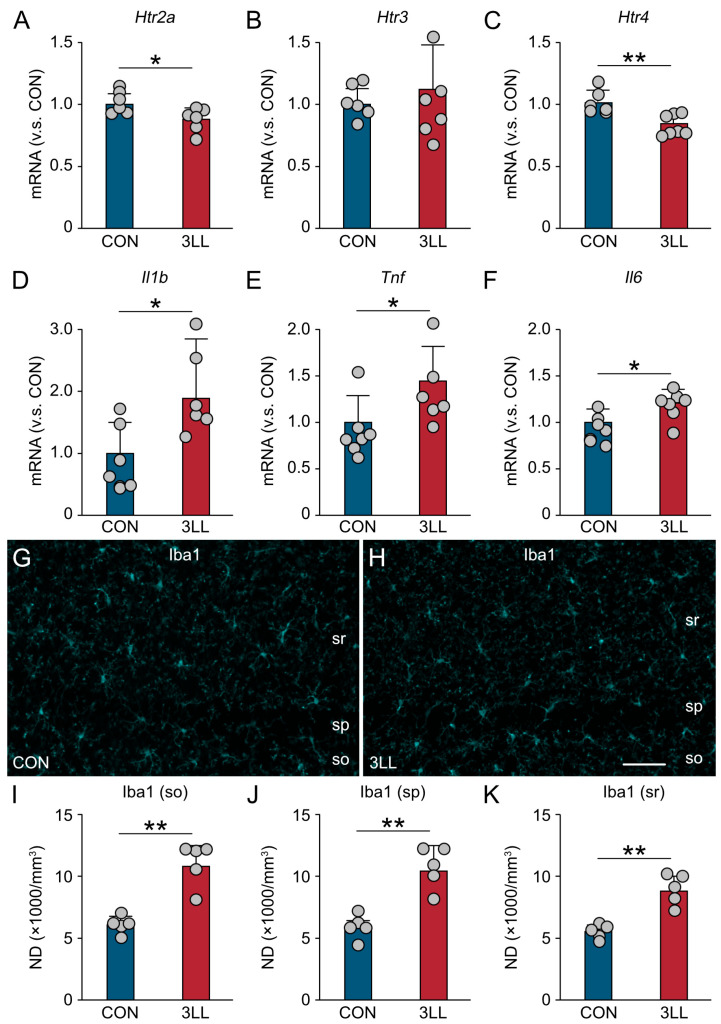
Alterations in serotonergic signaling, neuroinflammation, and microglial density in the hippocampus of 3LL mice. (**A**–**C**) Relative mRNA expression levels (normalized to CON) of serotonergic receptor genes in the hippocampus: *Htr2a* (**A**), *Htr3* (**B**), and *Htr4* (**C**). (**D**–**F**) Relative mRNA expression levels of pro-inflammatory cytokines *Il1b* (**D**), *Tnf* (**E**), and *Il6* (**F**). (**G**,**H**) Representative Iba1 immunostaining images of the CA1 region in the hippocampus of CON (**G**) and 3LL mice (**H**). (**I**–**K**) Numerical density (ND, ×1000/mm^3^) of Iba1^+^ microglia in the CA1region: stratum oriens (so) (**I**), stratum pyramidale (sp) (**J**), and stratum radiatum (sr) (**K**). Statistical differences were analyzed using Welch’s *t*-test. Data are presented as mean ± SD: RT-qPCR (CON, *n* = 6 mice; 3LL, *n* = 6 mice); immunohistochemistry (CON, *n* = 5 mice; 3LL, *n* = 5 mice). Each grey circle represents an individual animal. Asterisks denote statistical significance: * *p* < 0.05, ** *p* < 0.01. Scale bar in (**H**) = 30 µm [applies to (**G**,**H**)].

To investigate the interplay between serotonergic signaling and neuroinflammation, we analyzed the hippocampal expression of serotonergic receptors, pro-inflammatory cytokines, and microglial activation markers in 3LL mice using RT-qPCR (Figure 3). Our findings reveal a significant downregulation of serotonergic receptors, 5-HT receptor 2a (*Htr2a*), and 5-HT receptor 4 (*Htr4*) in 3LL mice when compared to CON mice. Interestingly, the expression of 5-HT receptor 3 (*Htr3*) remained unchanged (Figure 3A–C). Concurrently, we observed a notable increase in the expression of pro-inflammatory cytokine genes, specifically those encoding interleukin-1β (*Il1b*), TNF-α (*Tnf*), and IL-6 (*Il6*) (Figure 3D–F). These results collectively indicate a heightened neuroinflammatory state in the hippocampus of 3LL mice, which may be linked to the observed downregulation of serotonergic receptors.

Immunohistochemical analysis of ionized calcium binding adapter protein 1 (Iba1), a microglial marker, showed increased immunoreactivity in the cornu ammonis 1 (CA1) region of the hippocampus in 3LL mice (Figure 3G,H). Quantitative analysis revealed significantly higher densities of Iba1-positive microglia in the strata oriens, pyramidale, and radiatum (Figure 3I–K), suggesting localized microglial activation. Together, these findings suggest that lung cancer progression is associated with downregulation of specific serotonergic receptors and increased neuroinflammatory activity in the hippocampus.

### 2.4. Reduced Serotonergic Terminals and Enhanced Microglial Activation in the Hippocampus of 3LL Mice

**Figure 4:**
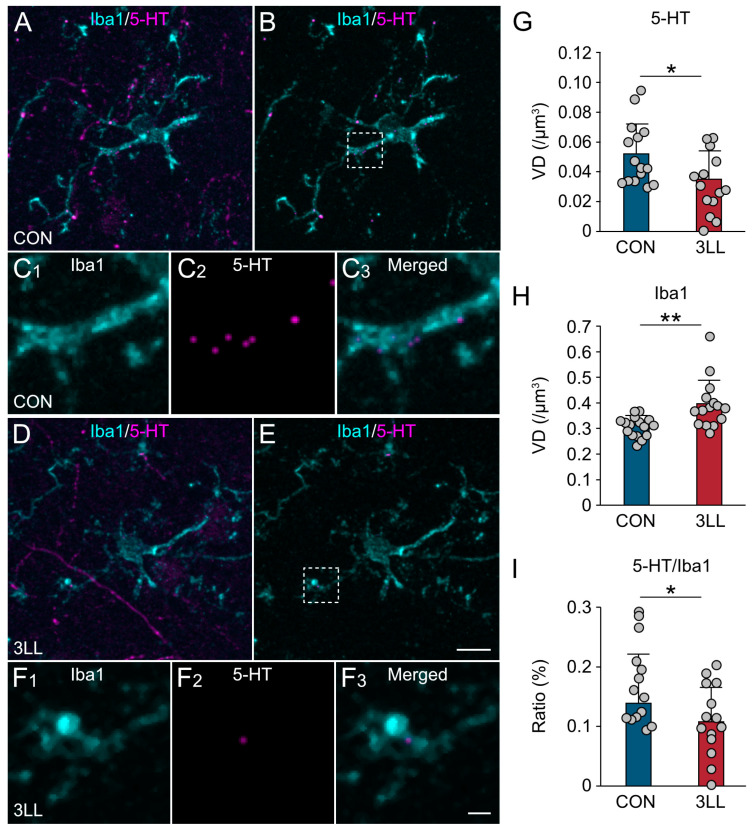
Reduction in serotonergic terminals in the hippocampus of 3LL mice. (**A**–**C**) Representative medium-power (**A**,**B**) and high-power (**C**) images of Iba1 (cyan) and 5-HT (magenta) immunostaining in the CA1 region of the hippocampus from CON mice. 5-HT^+^ terminals in contact with Iba1^+^ microglia were extracted by image subtraction (**B**,**C**). The boxed area in (**B**) is shown at a higher magnification in (**C**). (**D**–**F**) Representative medium-power (**D**,**E**) and high-power (**F**) images of Iba1 (cyan) and 5-HT (magenta) in the CA1 region of 3LL mice. Contacting 5-HT^+^ terminals were similarly extracted by image subtraction (**E**,**F**). The boxed area in (**E**) is enlarged in (**F**). (**G**–**I**) Quantification of voxel ratios (%) in the CA1 region: 5-HT^+^ voxels (**G**), Iba1^+^ voxels (**H**), and overlapping 5-HT^+^/Iba1^+^ voxels (**I**) relative to the total voxel count. Statistical differences were analyzed using Welch’s *t*-test. Data are presented as mean ± SD (CON, *n* = 14 sections; 3LL, *n* = 14 sections). Each grey circle represents a single section. Asterisks denote statistical significance: * *p* < 0.05, ** *p* < 0.01. Scale bar in (**E**) = 10 µm [applies to (**A**,**B**,**D**,**E**)]; scale bar in (**F_3_**) = 1 µm [applies to (**C_1_**_–**3**_,**F_1_**_–**3**_)].

To examine the impact of lung cancer on serotonergic synapses in the hippocampus, we performed a voxel-based quantification of 5-HT and Iba1 immunoreactivity (Figure 4). Overall, 5-HT signal intensity was moderately reduced, whereas Iba1 signal was elevated in 3LL mice (Figure 4A–F).

Quantitative voxel analysis revealed a significant reduction in 5-HT^+^ terminal density (Figure 4G) and a concomitant increase in Iba1^+^ microglial processes in 3LL mice (Figure 4H). Furthermore, the density of 5-HT^+^/Iba1^+^ synaptic contacts—indicative of serotonergic terminals closely associated with microglia—was reduced in 3LL mice compared to that in controls (Figure 4I). These findings suggest disrupted serotonergic innervation and elevated microglial activity in the hippocampus during lung cancer progression.

### 2.5. TBG Alleviates Anxiety-like Behavior and Cognitive Impairment in 3LL Mice

**Figure 5:**
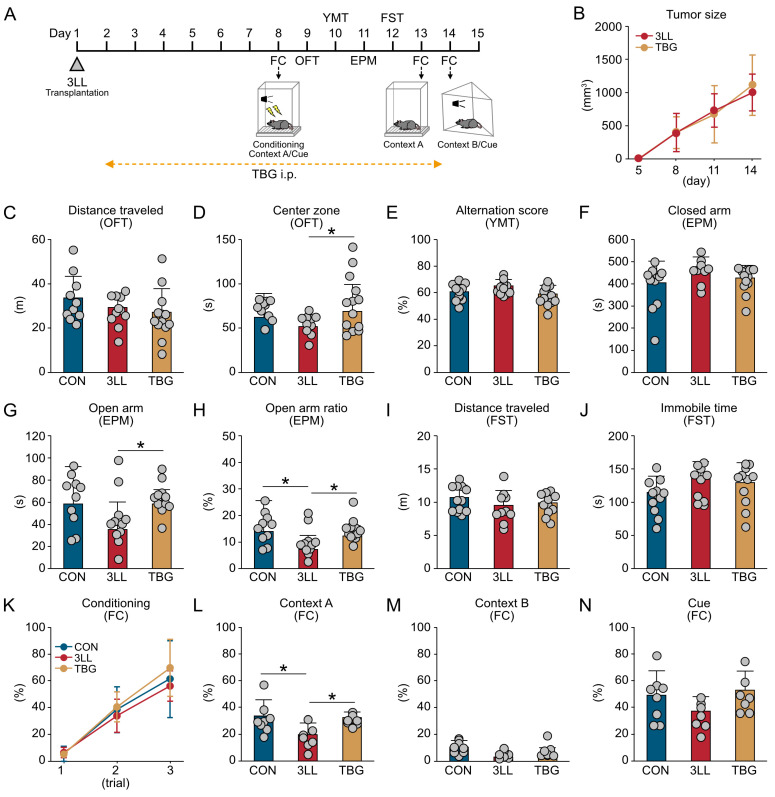
TBG restores emotional and cognitive function in 3LL mice. (**A**) Schematic timeline illustrating the experimental sequence: 3LL Lewis lung carcinoma cell transplantation, TBG administration, and the behavioral test battery, including the OFT, YMT, EPM test, FST, and FC test. (**B**) Tumor size progression in 3LL and TBG mice. (**C**,**D**) Total distance traveled (m) (**C**) and time spent in the center zone (s) (**D**) during the OFT. (**E**) Spontaneous alternation score (%) in the YMT. (**F**–**H**) Time spent in the closed arms (s) (**F**), open arms (s) (**G**), and open arm entry ratio (%) (**H**) in the EPM. (**I**,**J**) Distance traveled (m) (**I**) and immobility time (s) (**J**) in the FST. (**K**) Freezing time ratio (%) during the conditioning phase in context A. (**L**,**M**) Freezing time ratio (%) during memory recall in contexts A (**L**) and B (**M**). (**N**) Freezing time ratio (%) in response to the auditory cue (tone paired with foot shock). Statistical differences were analyzed using one-way analysis of variance (ANOVA) with Tukey’s honestly significant difference (HSD) post-hoc test. Data are presented as mean ± SD: OFT, YMT, EPM, and FST (CON, *n* = 11 mice; 3LL, *n* = 11 mice; TBG, *n* = 13 mice); FC (CON, *n* = 8 mice; 3LL, *n* = 7 mice; TBG, *n* = 7 mice). Each data point represents an individual animal. Asterisks denote statistical significance: * *p* < 0.05.

Given the observed serotonergic dysregulation, we assessed whether TBG, a non-hallucinogenic psychedelic analog and selective 5-HT_2A_ receptor agonist [19], could mitigate behavioral deficits in 3LL mice (Figure 5).

The experimental timeline is illustrated in Figure 5A. Tumor size did not differ significantly between untreated 3LL mice and those receiving TBG treatment (TBG mice), indicating that TBG did not affect tumor progression (Figure 5B). Locomotor activity, as assessed by total distance in the OFT, was unchanged across CON, 3LL, and TBG mice (Figure 5C). However, TBG mice spent significantly more time in the center zone than both CON and 3LL mice, indicating reduced anxiety-like behavior (Figure 5D).

In the YMT, no significant differences in spontaneous alternation scores were observed among groups, suggesting intact working memory (Figure 5E). In the EPM test, time spent in the closed arms was similar across groups (Figure 5F); however, TBG mice spent more time in the open arms than CON and 3LL mice (Figure 5G). Additionally, the open-to-closed arm time ratio was significantly higher in both CON and TBG mice than in 3LL mice (Figure 5H), further supporting an anxiolytic effect of TBG.

In the FST, no significant differences were found in total distance traveled or immobility time among the groups, indicating no major changes in depression-like behavior (Figure 5I,J). In the FC test, freezing behavior during the conditioning phase was similar across all groups (Figure 5K). However, 3LL mice exhibited impaired context-dependent memory recall, which was restored in TBG mice (Figure 5L). Freezing responses to context B and the auditory cue were not significantly different among the groups (Figure 5M,N). These findings indicate that TBG effectively mitigates anxiety-like behavior and ameliorates hippocampus-dependent contextual memory deficits in 3LL mice without discernible impact on general locomotion, working memory, or depression-like behavior.

### 2.6. Upregulation of Serotonergic Receptors and Suppression of Neuroinflammation in the Hippocampus of TBG Mice

**Figure 6:**
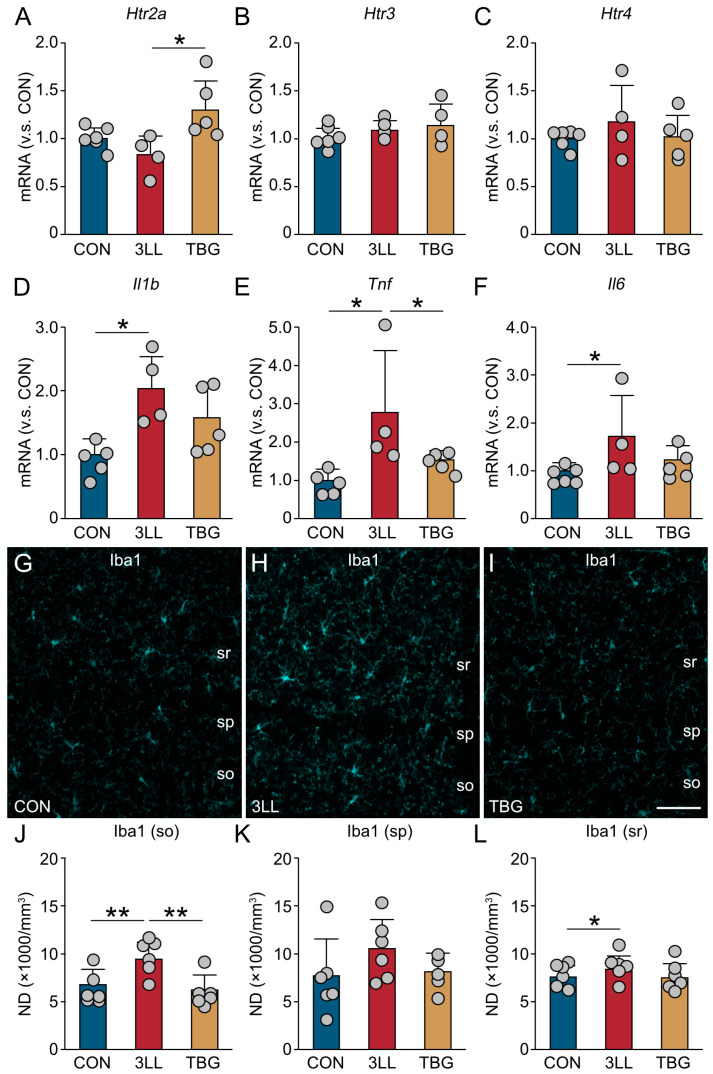
TBG reverses serotonergic and neuroinflammatory alterations in the hippocampus of 3LL mice. (**A**–**C**) Relative mRNA expression levels (normalized to CON) of serotonergic receptor genes in the hippocampus: *Htr2a* (**A**), *Htr3* (**B**), and *Htr4* (**C**) in 3LL and TBG mice. (**D**–**F**) Relative mRNA expression levels of pro-inflammatory cytokines *Il1b* (**D**), *Tnf* (**E**), and *Il6* (**F**) in the hippocampus of 3LL and TBG mice. (**G**–**I**) Representative Iba1 immunostaining images in the CA1 region of the hippocampus in CON (**G**), 3LL (**H**), and TBG (**I**) mice. (**J**–**L**) Numerical density (ND, ×1000/mm^3^) of Iba1^+^ microglia in the CA1region: stratum oriens (so) (**J**), stratum pyramidale (sp) (**K**), and stratum radiatum (sr) (**L**). Statistical differences were analyzed using one-way ANOVA with Tukey’s HSD post-hoc test. Data are presented as mean ± SD: RT-qPCR (CON, *n* = 6 mice; 3LL, *n* = 4 mice; TBG, *n* = 5 mice); immunohistochemistry (CON, *n* = 6 mice; 3LL, *n* = 6 mice; TBG, *n* = 6 mice). Each grey circle represents an individual animal. Asterisks denote statistical significance: * *p* < 0.05, ** *p* < 0.01. Scale bar in (**I**) = 30 µm [applies to (**G**–**I**)].

To further investigate the effects of TBG on hippocampal serotonergic signaling and neuroinflammation, we assessed the expression of genes related to serotonergic receptors and pro-inflammatory cytokines, as well as the density of Iba1^+^ microglia in the hippocampus (Figure 6).

Gene expression analysis revealed that the serotonergic receptor *Htr2a* was significantly upregulated in TBG mice compared to 3LL mice (Figure 6A), whereas *Htr3* and *Htr4* expression remained comparable across all groups (Figure 6B,C). Among the pro-inflammatory cytokines, *Il1b* and *Il6* expression were elevated in both 3LL and TBG mice relative to the controls (Figure 6D,F), while *Tnf* expression was significantly upregulated in 3LL mice but reduced in TBG mice to levels comparable to controls (Figure 6E).

Immunohistochemical analysis of Iba1 in the CA1 region showed moderately increased Iba1 immunoreactivity in 3LL mice (Figure 6G–I). Quantitative analysis confirmed elevated densities of Iba1^+^ microglia in the strata oriens, pyramidale, and radiatum of the CA1 region in 3LL mice, which were attenuated in TBG mice (Figure 6J–L). These findings suggest that TBG partially restores serotonergic receptor expression—particularly *Htr2a*—and attenuates lung cancer-induced neuroinflammation, as evidenced by reduced microglial activation and normalization of pro-inflammatory gene expression.

### 2.7. TBG Inhibits Alterations in Microglial Morphology and Gene Expression in the Hippocampus of 3LL Mice

Alterations in microglial process morphology are widely recognized as indicators of microglial activation, which may contribute to behavioral deficits [21]. To assess these changes, we conducted three dimensional (3D) reconstructions of Iba1^+^ microglia using Neurolucida (Figure S1). In 3LL mice, Iba1^+^ microglial processes exhibited reduced complexity and decreased total length compared to the controls, whereas microglia in TBG mice retained a higher degree of structural complexity (Figure S1A–C). No significant differences were observed in cell body area or the number of primary processes among groups (Figure S1D,E). However, 3LL mice showed a marked reduction in the number of process nodes, process ends, and total process length compared to both CON and TBG mice (Figure S1F–H). Additionally, mean process length and process volume were reduced in both 3LL and TBG mice relative to the controls (Figure S1I,J), while the convex hull volume was significantly smaller in 3LL mice than in CON and TBG mice (Figure S1K).

Further analysis of microglial architecture using 3D reconstruction revealed that first-order process length and node number were significantly reduced in 3LL mice compared to those in the controls (Figure S2A,B). However, second- and third-order process lengths and node numbers were comparable across all groups (Figure S2C–F). Sholl analysis [22] revealed no significant differences in the number of intersections or process length at 10 μm from the soma (Figure S2G,H). At 20 μm, both metrics were significantly reduced in 3LL mice compared to those in CON and TBG mice (Figure S2I,J), and at 30 μm, they were lower in 3LL mice than in CON mice (Figure S2K,L).

To complement the morphological findings, we examined the expression of genes associated with microglial activation using RT-qPCR (Figure S3). We observed an elevated expression of the gene encoding Iba1 (*Aif1*) in 3LL mice compared to the controls (Figure S3A), while transmembrane protein 119 (*Tmem119*) expression remained consistent across all groups (Figure S3B). Notably, purinergic receptor P2Y12 (*P2ry12*) showed a significantly higher expression in 3LL mice than in both CON and TBG mice (Figure S3C), whereas purinergic receptor P2Y13 (*P2ry13*) expression was similar across groups (Figure S3D). Furthermore, cluster of differentiation 68 (*Cd68*) expression was elevated in 3LL mice relative to the controls (Figure S3E), while cluster of differentiation 33 (*Cd33*) expression remained unchanged (Figure S3F). Additionally, the expression of C-X3-C motif chemokine receptor 1 (*Cx3cr1*) and complement component 1q *(C1q*) was higher in 3LL mice compared to CON mice (Figure S3G,H). Collectively, these findings suggest that TBG treatment may partially inhibit lung cancer-induced microglial activation in the hippocampus.

### 2.8. TBG Suppresses Neuroinflammatory Gene Expression in BV-2 Microglia Exposed to 3LL Cell-Conditioned Medium

**Figure 7:**
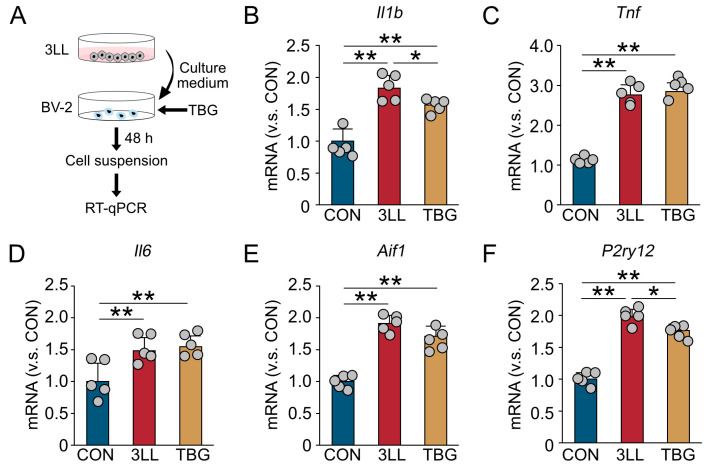
TBG attenuates neuroinflammatory gene expression in BV-2 microglia exposed to 3LL cell-conditioned medium in vitro. (**A**) Schematic timeline of the experimental design: BV-2 microglia were treated with control medium (CON), conditioned medium from 3LL cells (3LL), or 3LL-conditioned medium supplemented with TBG (TBG). After 48 h of incubation, BV-2 cell suspensions were collected for RT-qPCR analysis. (**B**–**D**) Relative mRNA expression levels (normalized to CON) of pro-inflammatory cytokine genes in BV-2 cells *Il1b* (**B**), *Tnf* (**C**), and *Il6* (**D**) across CON, 3LL, and TBG treatment groups. (**E**,**F**) Relative mRNA expression levels (normalized to CON) of microglial activation markers *Aif1* (**E**) and *P2ry12* (**F**) in the same treatment groups. Statistical differences were analyzed using one-way ANOVA with Tukey’s HSD post-hoc test. Data are presented as mean ± SD from RT-qPCR (CON, *n* = 5 experiments; 3LL, *n* = 5 experiments; TBG, *n* = 5). Each grey circle represents a single experiment. Asterisks denote statistical significance: * *p* < 0.05, ** *p* < 0.01.

To directly assess the anti-inflammatory effects of TBG on microglia, we conducted an in vitro assay using the BV-2 microglial cell line (Figure 7). BV-2 cells were exposed to conditioned medium derived from 3LL cells, with or without co-treatment with TBG. After 48 h of incubation, gene expression in BV-2 cells was analyzed using RT-qPCR (Figure 7A).

Exposure to 3LL cell-conditioned medium alone significantly upregulated the expression of pro-inflammatory cytokines *Il1b*, *Tnf*, and *Il6* in BV-2 cells (Figure 7B–D). Notably, co-treatment with TBG effectively suppressed the elevated expression of *Il1b* (Figure 7B). Although *Tnf* and *Il6* levels remained elevated in the presence of TBG, their induction was not further exacerbated, suggesting a partial modulatory effect (Figure 7C,D).

In addition, the expression of microglial activation markers *Aif1* and *P2ry12* was significantly increased in BV-2 cells treated with 3LL-conditioned medium alone; this upregulation was attenuated by TBG co-treatment (Figure 7E,F). Collectively, these findings demonstrate that TBG suppresses the expression of genes associated with neuroinflammation and microglial activation in response to tumor-derived factors, supporting its potential as a modulator of cancer-induced neuroimmune dysregulation.

## 3. Discussion

In this study, we conducted a comprehensive behavioral test battery to characterize a rodent model of CRCI and evaluate the therapeutic potential of TBG, a non-hallucinogenic psychedelic analog. Our initial observations revealed comparable total distance traveled during the OFT between CON and 3LL mice, indicating that motor dysfunction was not a confounding factor in our cancer model. However, 3LL mice showed a significant reduction in time spent in the open arms of the EPM, indicating anxiety-like behavior. Furthermore, performance in the FC test demonstrated an impaired contextual memory, a cognitive process reliant on hippocampal integrity. These behavioral deficits are consistent with clinical reports of CRCI in patients with various malignancies, including colorectal, lung, breast, ovarian, prostate, and testicular cancers [23].

Importantly, anxiety and cognitive dysfunction can manifest in patients with cancer even before the initiation of anti-cancer treatments [23,24,25]. Although most research has focused on chemotherapy-induced side effects, commonly termed “chemobrain” [26,27], studies investigating the direct impact of peripheral cancer on cognitive abilities are limited, and comprehensive behavioral test batteries for evaluating cognitive function in mice with transplanted tumors are scarce [28,29]. Our findings support the validity of the 3LL cell transplantation model for studying CRCI and highlight its relevance in replicating behavioral deficits observed in patients with cancer.

A potential mechanism underlying CRCI involves a cancer-associated upregulation of enzymes such as IDO and TDO, which drive systemic tryptophan catabolism into kynurenine [9,30]. Consistent with this, we observed decreased tryptophan levels in both the blood and hippocampus of 3LL mice. Our findings are particularly relevant to cognitive function, as kynurenine pathway metabolites are neuroactive and can directly influence neuronal function. For example, quinolinic acid, a downstream kynurenine metabolite, acts as an N-methyl-D-aspartate receptor agonist and can induce excitotoxicity at elevated levels, a key contributor to neuronal damage and cognitive decline [31]. Recent research involving cisplatin suggests that certain kynurenine metabolites can cross the BBB and elevate kynurenic acid levels, which are associated with cognitive impairment [32]. The resulting imbalance between excitatory and inhibitory metabolites may disrupt neuronal processing and contribute to CRCI. Future research should prioritize elucidating the specific roles of different kynurenine metabolites in CRCI and exploring potential therapeutic interventions that target this pathway to mitigate cognitive decline in patients with cancer.

Our data further reveal markedly reduced densities of 5-HT^+^ terminals and the downregulation of serotonergic receptor genes in the hippocampus of 3LL mice. These findings are in line with previous studies showing that systemic tryptophan depletion can impair central 5-HT synthesis, thereby increasing the risk of mood disturbances and depression [33]. Compelling clinical evidence also highlights the significant role of serotonergic signaling in mood disorders through its interaction with immune cells [34]. Microglia, the brain’s resident immune cells, maintain brain homeostasis through 5-HT-mediated suppression, thereby preventing excessive neuroinflammation [35]. In this study, we observed a corresponding increase in the density of Iba1^+^ microglia and the expression of pro-inflammatory cytokine genes in the hippocampus of 3LL mice. Given the hippocampus’s pivotal role in emotional regulation and the involvement of microglia in anxiety-like behaviors, we propose that peripheral cancer triggers microglial activation, contributing to depressive and anxiety-like behaviors observed in CRCI.

Notably, TBG significantly attenuated anxiety-like behavior in 3LL mice, suggesting its potential as a novel anxiolytic agent for managing cancer-related mood disturbances. While hallucinogenic psychedelics have been extensively explored for psychological distress (e.g., anxiety and depression) in patients with cancer, particularly in palliative settings [36], our results underscore the therapeutic promise of non-hallucinogenic alternatives. Similar to classic psychedelics, TBG targets serotonergic receptors, particularly the 5-HT_2A_ receptor [37], but without inducing hallucinogenic effects, potentially due to differences in signaling pathways or receptor activation profiles. The anxiolytic properties of serotonergic compounds are supported by preclinical models [38] and clinical trials showing reduced anxiety and depression and improved quality of life in patients with cancer undergoing psilocybin therapy [39]. Notably, high-dose psilocybin has demonstrated sustained reductions in anxiety and depression for several months in patients with advanced-stage cancer [40]. Given that 5-HT_2A_ receptor activation is considered central to the therapeutic effects of psychedelics, our findings strongly suggest that TBG exerts its anxiolytic effects through the modulation of these receptors, offering a potential therapeutic benefit without the psychoactive challenges associated with hallucinogens.

A particularly significant finding of this study is the reversal of memory impairment by TBG treatment in 3LL mice. Corroborating this cognitive improvement, we observed that TBG treatment reduced the density of Iba1^+^ microglia and suppressed the gene expression of pro-inflammatory cytokines in the hippocampus. This observation aligns with the growing evidence that a substantial proportion of patients with cancer exhibit cognitive decline even prior to initiating anti-cancer therapies [25]. A leading hypothesis attributes pre-treatment CRCI to neuroinflammation, proposing that tumor-derived inflammatory mediators enter systemic circulation. This systemic inflammation, potentially augmented by peripheral immune activation and BBB alterations, can trigger and sustain neuroinflammation, disrupting brain homeostasis and leading to cognitive deficits such as memory impairment [41].

The shared 5-HT_2A_ agonism among various psychedelics suggests that their anti-inflammatory effects may stem from common biochemical pathways [42]. Emerging preclinical data highlight the anti-inflammatory potential of serotonergic psychedelics, including psilocybin, LSD, and DMT, within the central nervous system. For instance, psilocin (the active metabolite of psilocybin) modulates microglial activity in a 5-HT_2_ receptor-dependent manner, inhibiting microglial phagocytosis and reactive oxygen species and nitric oxide release [43]. Furthermore, psilocybin can modulate the expression of inflammatory genes, such as *Il17* [44]. Similarly, DMT modulates immune responses via 5-HT receptors, suppressing inflammatory cytokine release from dendritic cells [45]. Collectively, these findings support the hypothesis that TBG’s interaction with the 5-HT_2A_ receptor likely mediates the reduction in neuroinflammatory markers observed in our 3LL model.

TBG may facilitate cognitive recovery not only by reducing neuroinflammation but also by enhancing synaptic plasticity and neurogenesis, both of which are fundamental processes underlying learning and memory. By alleviating the inflammatory burden on neural circuits, TBG could help re-establish the functional integrity of brain regions compromised by CRCI. Serotonergic psychedelics induce neuroplastic changes independently of their anti-inflammatory effects [46], suggesting that TBG may contribute directly to memory restoration through structural and functional modifications at the synaptic level. In addition, psychedelics modulate large-scale brain network connectivity, potentially enabling a “reset” of maladaptive neural circuits associated with cognitive dysfunction [47]. Thus, the cognitive improvements observed with TBG likely arise from a multifaceted mechanism involving the attenuation of neuroinflammation, enhancement of neuroplasticity, and possibly indirect benefits through the reduction of anxiety-like behaviors.

Further supporting the role of TBG in modulating neuroinflammation, our study showed that TBG treatment normalized the aberrant hippocampal microglial morphology in 3LL mice. These in vivo findings were reinforced by in vitro experiments, where TBG reversed the expression of genes related to neuroinflammation and microglial activation, i.e., *Il1b*, *Aif1*, and *P2ry12*, in BV-2 cells exposed to 3LL-conditioned media. Although 3LL cells are widely used, the specific influence of their secreted factors on BV-2 cytokine production had not been directly examined, adding a novel dimension to our in vitro results. Previous research has reported that serotonergic compounds, such as (±)-2,5-dimethoxy-4-iodoamphetamine, exert anti-inflammatory effects by selectively modulating 5-HT_2A_ receptor signaling, including the suppression of arginase 1 expression through functionally selective mechanisms [48]. This suggests that TBG may similarly modulate serotonergic pathways—either directly or indirectly—thereby influencing microglial activation in the context of cancer-induced neuroinflammation.

Several limitations warrant consideration when interpreting the findings of this study. First, the current work is constrained by its primary focus on the hippocampus. Given the complex and distributed nature of cognitive and emotional regulation, future studies should investigate other key brain regions, such as the prefrontal cortex and amygdala. Second, longitudinal studies are essential to evaluate the durability of TBG’s therapeutic effects and to determine its long-term safety profile. Third, our study exclusively used male mice, which may limit the generalizability of the findings. Sex-based biological differences can influence physiological and behavioral responses [49]; therefore, future studies should include both male and female subjects to fully assess potential sex-specific effects of TBG. Finally, the precise molecular and cellular mechanisms underlying TBG’s actions remain to be elucidated. Investigations into its pharmacokinetics, receptor binding profiles, and downstream signaling cascades will provide valuable insights into its therapeutic potential.

In conclusion, our study presents compelling evidence that TBG ameliorates cognitive and emotional impairments in a mouse model of CRCI. These therapeutic effects appear to be mediated, at least in part, by the restoration of serotonergic signaling and the suppression of neuroinflammatory processes within the hippocampus. These findings not only underscore the potential of TBG as a neuroprotective agent, but also highlight the critical role of serotonergic and inflammatory pathways in the pathophysiology of CRCI. Ultimately, addressing these gaps will be vital for translating these preclinical findings into clinical applications and positioning TBG as a promising candidate in the development of novel interventions for CRCI.

## 4. Materials and Methods

### 4.1. Animals

A total of 78 male C57BL/6J mice (CLEA Japan, Tokyo, Japan) were used in this study. All animals were housed under a 12 h light–dark cycle with ad libitum access to food and water. All procedures were approved by the Committee of Ethics on Animal Experiments in the Graduate School of Medical Sciences, Kyushu University (Approval No: A22-221-0, approved on 1 April 2022 and Approval No: A24-060-2, approved on 1 April 2024), and conducted in accordance with the Animal Research: Reporting of In Vivo Experiments guidelines [50]. All efforts were made to minimize the number of animals used and reduce their suffering.

### 4.2. Cell Line

The 3LL cell line was obtained from the Cell Resource Center for Biomedical Research, Institute of Development, Aging, and Cancer, Tohoku University. Cells were cultured in Dulbecco’s modified Eagle’s medium supplemented with 10% fetal bovine serum (FBS) and penicillin–streptomycin and maintained at 37 °C in a humidified incubator with 5% CO_2_.

### 4.3. Cancer Transplantation

To generate cancer-bearing mice, 3LL cells [5 × 10^5^ cells in 500 μL phosphate-buffered saline (PBS)] were injected subcutaneously into the dorsal flank. Tumor volume was measured every 2–3 days using a digital caliper and calculated as:

Tumor volume (mm^3^) = 0.5 × length × width^2^

In compliance with institutional animal welfare guidelines, mice with tumor volumes exceeding 2000 mm^3^ were excluded from the analysis to prevent undue pain and distress.

### 4.4. Drug Administration and Animal Groups

Cancer-bearing mice received daily intraperitoneal (i.p.) injections of either TBG (30 mg/kg; Merck, Billerica, MA, USA) or vehicle (saline, 5 mL/kg) from day 1 to day 14.

Animals were divided into three groups: control mice receiving either vehicle or no treatment (CON mice); 3LL cell-transplanted cancer-bearing mice receiving either vehicle or no treatment (3LL mice); and 3LL cell-transplanted cancer-bearing mice receiving TBG treatment (TBG mice).

### 4.5. Behavioral Test Battery

A total of 16 mice were used to examine the effects of cancer transplantation using the behavioral test battery (*n* = 8 for CON mice, *n* = 8 for 3LL mice). A total of 35 mice were used to examine the effects of TBG using the behavioral test battery (*n* = 11 for CON mice, *n* = 11 for 3LL mice, *n* = 13 for TBG mice).

On day 9, locomotor activity was evaluated using the OFT. The mice were placed in a square open field chamber (50 × 50 × 50 cm; Muromachi Kikai, Tokyo, Japan) for 10 min and allowed to move freely. The center area (30 × 30 cm) was illuminated at 100 lx. An entry was recorded when 50% of the animal’s body entered the center area. The distance traveled (m) and time (s) spent in the center and outer zones were automatically measured using a computer-assisted data acquisition system, ANY-maze (Stoelting, Wood Dale, IL, USA).

On day 10, short-term working memory was evaluated using the YMT. The mice were placed in a Y-shaped maze apparatus with three opaque arms (10 × 30 × 10 cm; Muromachi Kikai), spaced 120° apart, for 8 min and allowed to move freely. The center of the field was illuminated at 100 lx. An entry was defined as the crossing of more than 80% of the animal’s body (excluding the tail) into an arm, which was automatically detected by the ANY-maze (Stoelting). The following formula was used to measure short-term working memory:

Spontaneous alternation score (%) = [(number of alternations)/(total number of arm entries − 2)] × 100

On day 11, anxiety-like behavior was examined using the EPM test. The mice were placed in an EPM apparatus that consisted of two open arms (30 × 6 × 15 cm), two closed arms (30 × 6 cm), and a center platform (6 × 6 cm; Muromachi Kikai). The apparatus was raised 40 cm above the floor, and the center of the maze was illuminated at 100 lx. The mice were placed in the elevated plus maze for 10 min and allowed to move freely. The time (s) spent in the open and closed arms was automatically measured by the ANY-maze (Stoelting), and entry was recorded when 50% of the animal’s body entered each arm.

Long-term contextual memory was evaluated using the FC test. On day 8, the mice were placed in a square chamber (18 × 18 × 40 cm; context A) with a grid floor, connected to a shock generator scrambler (MFS-01, Muromachi Kikai). During the conditioning session, the mice could move freely in the chamber for 2 min. Subsequently, white noise was used as a conditioned stimulus (CS) for 30 s, with a foot shock (0.5 mA, 2 s) delivered as an unconditioned stimulus (US) during the final 2 s of the CS. The mice were subjected to two additional CS-US pairings at 2 min intervals. On day 13, the mice were placed back in the conditioned square chamber (context A) for 6 min as a contextual fear test. The following day, the mice were placed in a triangular chamber (18 × 18 × 18 cm; context B) with a flat floor for 6 min for the cued fear test. In this chamber, the mice could move freely, and the CS was applied during the last 3 min of the test period. Freezing behavior (defined as a complete lack of motion for a minimum of 1 s) was automatically measured by ANY-maze (Stoelting).

### 4.6. Perfusion Fixation and Sectioning

For immunohistochemistry, 29 mice (*n* = 11 for CON mice, *n* = 12 for 3LL mice, *n* = 6 for TBG mice) were anesthetized with an overdose of sodium pentobarbital (120 mg/kg, i.p.) and perfused transcardially with PBS, followed by a fixative solution consisting of a mixture of 4.0% paraformaldehyde (PFA) and 0.05% glutaraldehyde in 0.1 M phosphate buffer (PB, pH 7.4). The brains were post-fixed in situ for 1–2 h at room temperature and subsequently removed from the skull. Brain blocks were divided along the midline and cut into 40 μm-thick coronal sections on a vibrating blade microtome (VT1000S; Leica Microsystems, Wetzlar, Germany). To avoid deformation of the sections, they were processed free-floating with extreme caution.

### 4.7. Measurement of Tryptophan and Kynurenine Concentrations

A total of 13 mice (*n* = 6 for CON mice, *n* = 7 for 3LL mice) were used for the measurement of tryptophan and kynurenine concentrations. For the measurement of tryptophan and kynurenine levels in serum, a blood sample was collected from the tail vein, and serum was collected and stored at −20 °C. For the measurement of tryptophan and kynurenine levels in the brain, the animals were anesthetized with an overdose of sodium pentobarbital (120 mg/kg, i.p.) and perfused transcardially with PBS; the brains were subsequently removed from the skull. Tryptophan and kynurenine concentrations were measured by an ELISA kit (Immundiagnostik AG, Bensheim, Germany) according to the manufacturer’s protocol.

### 4.8. Voxel Counting Analysis of 5-HT^+^ Terminals

To quantitatively estimate the contact between Iba1^+^ microglia and 5-HT^+^ terminals, we applied the voxel counting tool in ImageJ 1.53 (National Institutes of Mental Health, Bethesda, MD, USA). A total of 10 mice (*n* = 5 for CON mice, *n* = 5 for 3LL mice) were used for the voxel counting analysis of 5-HT. Three sections per animal were processed for fluorescent staining of Iba1 and 5-HT. One stack of optical sections containing the CA1 was captured using an optical sectioning microscope (Apotome 2, Carl Zeiss) equipped with an oil-immersion objective lens (×63, NA 1.40). Images for each channel (cyan, 5-HT; green, Iba1) were processed separately to optimize the detection of Iba1^+^ cells or 5-HT^+^ terminals. The contrast for the cyan channel was corrected using the “enhance contrast” function (saturated pixels = 0.1%, normalized, all slices processed), and the background was reduced using the “subtract background” function (rolling ball radius = 2 pixels). The contrast for the green channel was adjusted using the “enhance contrast” function (saturated pixels = 0.1%, normalized, all slices processed), and background noise was reduced using the “subtract background” function (rolling ball radius = 10 pixels). All images were then thresholded for segmentation using the “make binary” function (auto threshold, “Otsu” method). The images were filtered through a 3D median filter (radius = 2 pixels in every dimension). To estimate the contact between 5-HT^+^ terminals and Iba1^+^ processes, we defined the granule cell layer using the 3D region of interest tool. The colocalized voxels (voxels double-positive for 5-HT and Iba1) were obtained using the image calculator (operation “multiply”). The voxel numbers were then measured using the voxel counter, and the voxel density (VD) was estimated using the following formula:

VD (/μm^3^) = Σ*Pov*/VOI

where Σ*Pov* represents the number of positive voxels and VOI represents the volume of the region of interest.

### 4.9. Optical Disector Analysis

A total of 15 mice (*n* = 5 for CON mice, *n* = 5 for 3LL mice, *n* = 5 for TBG mice) were used to count Iba1^+^ microglia by the optical disector [51]. A total of 18 mice (*n* = 6 for CON mice, *n* = 6 for cancer-bearing mice, *n* = 6 for cancer-bearing mice injected with TBG) were used to count Iba1^+^ microglia by the optical disector. Two sections per animal were selected and processed for immunostaining. One optical section stack was obtained from the CA1 region of the hippocampus with a structured illumination microscope (Axio Imager M2 with Apotome.2; Carl Zeiss, Oberkochen, Germany) using a dry objective lens (×20, NA 0.8). The pixel size (0.65 × 0.65 μm) and z-interval (1.0 μm) were optimized for rational and efficient cell counting.

The top and bottom of the sections, known as guard zones, were excluded from the cell counting, since sectioning can extract parts of the whole cells close to the cutting plane. These extracted cells are known as “lost caps” [52]. Considering the guard zone, the start and end points of the optical disector height were set at approximately 2.0 μm from the surfaces of the mounted tissue. The optical section at the starting point was used as a look-up, and the remaining sections were used as reference sections.

An unbiased counting frame [53] was superimposed onto the optical section stack with the Blend Images plugin of ImageJ 1.53 (National Institutes of Mental Health). All cells in the look-up section were disregarded and marked with a single color. Cells exclusively included in the reference sections were counted and marked with a different color. The numerical density (ND) was estimated using the following formula:

ND (×1000/mm^3^) = Σ*Q*^−^/[*h* × *a*(*fra*)/SV]

where Σ*Q*^−^ is the number of disector-counted cells in the subgranular zone, *h* is the optical disector height, *a*(*fra*) is the area of the counting frame, and SV is the volumetric shrinkage factor. In this study, the average SV value for Vectashield-mounted preparations (0.65) was used [54].

### 4.10. Morphometric Analysis

A total of 18 mice (*n* = 6 for CON mice, *n* = 6 for 3LL mice, *n* = 6 for TBG mice) were used to quantify morphological changes in Iba1^+^ microglia. From each animal, two sections were selected and processed for immunostaining. In total, 137 cells were analyzed. Optical section stacks were obtained from the CA1 region with a structural illumination microscope (Axio Imager.M2 with Apotome.2; Carl Zeiss) using an oil-immersion objective lens (× 63, NA 1.40). We used Neurolucida software version 2020 (MBF Bioscience, Williston, VT, USA) to carry out the 3D reconstruction of the microglia. The data sets were quantitatively analyzed using Neurolucida Explorer (MBF Bioscience). The following 3D morphometric parameters were obtained:

(1) Number of primary processes: The total number of primary processes.
(2) Number of nodes: The total number of process nodes.
(3) Number of ends: The total number of process ends.
(4) Total process length: The sum of the lengths of all process branches.
(5) Mean process length: The average length of all process branches.
(6) Process volume: The total volume of all process branches.
(7) Convex hull: The area of the convex polygon formed by connecting the tips of the most distal processes.
(8) Cell body: the area of the cell body.
(9) Length of 1st/2nd/3rd processes: The total length of the 1st, 2nd, and 3rd process branches, respectively.
(10) Number of nodes in 1st/2nd/3rd processes: The total number of nodes in the 1st, 2nd, and 3rd processes, respectively.
(11) Number of intersections (Sholl analysis): The number of intersections between processes and concentric circles at increasing distances from the cell body [22].
(12) Process length (Sholl analysis): The cumulative length of processes intersecting concentric circles at increasing distances from the cell body.

### 4.11. Culture of BV-2 Microglia

The BV-2 cells, a murine microglial cell line, were cultured in Dulbecco’s modified Eagle’s medium containing 10% FBS and penicillin–streptomycin. The cells were maintained at 37 °C and 5% CO_2_ in a humidified incubator. BV-2 cells were exposed to conditioned medium derived from 3LL cells, with or without co-treatment with TBG (10 µM). After 48 h of incubation, gene expression in BV-2 cells was analyzed.

### 4.12. RT–qPCR

A total of 27 mice (*n* = 14 for CON mice, *n* = 7 for 3LL mice, and *n* = 6 for TBG mice) were used for RT–qPCR. After the administration of an overdose of sodium pentobarbital (120 mg/kg, i.p.), deeply anesthetized animals were transcardially perfused with ice-cold PBS. Dorsal hippocampal tissues were collected from the brain on ice and kept in Gene Keeper RNA and DNA stabilization solution (NIPPON GENE, Tokyo, Japan).

Tissues were homogenized using a BioMasher homogenizer (Nippi, Tokyo, Japan) in lysis buffer. Total RNA was extracted from the dorsal hippocampus using the FastGene RNA Basic kit (Nippon Genetics, Tokyo, Japan). RNA quantity and purity were assessed using a NanoDrop 2000 spectrophotometer (Thermo Fisher Scientific, Waltham, MA, USA), with 260/280 ratios between 1.8 and 2.0, and 260/230 ratios between 2.0 and 2.2. For cDNA synthesis, 500 ng of total RNA was reverse-transcribed using ReverTra Ace qPCR RT Master Mix with gDNA Remover (Toyobo, Osaka, Japan) under the following cycling conditions: 37 °C for 15 min for gDNA removal, followed by 98 °C for 5 min to inactivate the enzyme. cDNA samples were aliquoted and stored at −30 °C, avoiding repeated freeze–thaw cycles.

RT–qPCR was performed on a QuantStudio 3 Real-Time PCR System (Thermo Fisher Scientific) using PowerUp™ SYBR™ Green Master Mix (Applied Biosystems) and gene-specific primers. All reactions were performed under identical thermal cycling conditions. Each 20 µL reaction contained 10 µL of SYBR Green Master Mix, 2 µL of each primer (final concentration 0.5 µM each), and 8 µL of cDNA template. The thermal cycling protocol included an initial holding stage at 50 °C for 2 min and 95 °C for 10 min, followed by 40 cycles of denaturation at 95 °C for 15 s and annealing/extension at 60 °C for 1 min. All reactions were run in doublets. Expression levels of genes of interest were compared to the reference gene, glyceraldehyde-3-phosphate dehydrogenase (*Gapdh*), and presented as a ratio relative to the reference gene. Data were analyzed using QuantStudio Design & Analysis Software v2.7.0 (Thermo Fisher Scientific). Amplification efficiencies for each primer set were determined by standard curve analysis using five serial dilutions of cDNA, and expression levels were calculated using the Pfaffl method for relative quantification [55]. In this study, we examined the expression levels of the following genes using RT–qPCR. The primer sequences are listed in Table 1.

(1) *Tph2*.
(2) *Ido1*.
(3) *Ido2*.
(4) *Tdo2*.
(5) *Htr2a*.
(6) *Htr3*.
(7) *Htr4*.
(8) *Il1b*.
(9) *Tnf*.
(10) *Il6*.
(11) *Aif1*.
(12) *Tmem119*.
(13) *P2ry12*.
(14) *P2ry13*.
(15) *Cd68*.
(16) *Cd33*.
(17) *Cx3cr1*.
(18) *C1q*.
(19) *Gapdh*.

### 4.13. Statistical Analysis and Illustration Preparations

All data sets were analyzed using OriginPro 2016 (OriginLab, Northampton, MA, USA). Statistical differences were analyzed using one-way ANOVA with Tukey’s HSD post-hoc test. Significant differences were considered to occur when a *p* value of <0.05 was obtained. Statistical details are summarized in Tables S1–S3. To prepare figures, selected images were processed using Affinity Photo V1 (Serif Europe, Nottinghamshire, UK). Only brightness and contrast were uniformly adjusted across the entire image frame; no part of any frame was enhanced or modified in any way.

**Table 1:** Sequences of the primers used in this study.

**Gene**	**Forward**	**Reverse**
*Tph2*	CCACCATTGTGACCCTGAATCC	ATGAGGACTCGGTGAGAGCATC
*Ido1*	GCAGACTGTGTCCTGGCAAACT	AGAGACGAGGAAGAAGCCCTTG
*Ido2*	GACAGTCTTGGTGGAGAAGGCA	ATCCTGGATGGAGAGTCTCAGC
*Tdo2*	CATGCTCAAGGTGATAGCTCGG	GGAAGCCTGATGCTGGAGACAG
*Htr2a*	CCTGATGTCACTTGCCATAGCTG	CAGGTAAATCCAGACGGCACAG
*Htr3*	CACACTCCTTCTGGGATACTCAG	GATGGTCTCAGCGAGGCTTATC
*Htr4*	TGCTCACGTTCCTTGCAGTGGT	GTCAGCAAAGGCGAGAGACACA
*Il1b*	TGGACCTTCCAGGATGAGGACA	GTTCATCTCGGAGCCTGTAGTG
*Tnf*	GGTGCCTATGTCTCAGCCTCTT	GCCATAGAACTGATGAGAGGGAG
*Il6*	TACCACTTCACAAGTCGGAGGC	CTGCAAGTGCATCATCGTTGTTC
*Aif1*	TCTGCCGTCCAAACTTGAAGCC	CTCTTCAGCTCTAGGTGGGTCT
*Tmem119*	ACTACCCATCCTCGTTCCCTGA	TAGCAGCCAGAATGTCAGCCTG
*P2ry12*	CATTGACCGCTACCTGAAGACC	GCCTCCTGTTGGTGAGAATCATG
*P2ry13*	TGGCATCAGGTGGTCAGTCACA	TTGTGCCTGCTGTCCTTACTCC
*Cd68*	GGCGGTGGAATACAATGTGTCC	AGCAGGTCAAGGTGAACAGCTG
*Cd33*	TGCAGAACATCACAATGAGAAAC	GAAAGGACCATCCAGCTCAA
*Cx3cr1*	TCTGGACTCACTACCTCATCAG	TCCGGTTGTTCATGGAGTTGG
*C1q*	ATGGAGACCTCTCAGGGATG	ATACCAGTCCGGATGCCAGC
*Gapdh*	CATCACTGCCACCCAGAAGACTG	ATGCCAGTGAGCTTCCCGTTCAG

## Data Availability

The data supporting the findings of this study are contained within the article and Supplementary Materials.

## Supplementary Materials

The following supporting information can be downloaded at: https://www.mdpi.com/article/10.3390/ijms26157519/s1.
