# Supplementary material for: Tabernanthalog, a Non-Hallucinogenic Psychedelic, Alleviates Cancer-Induced Cognitive Deficits via Serotonergic Pathways
Source: Int J Mol Sci. 2025 Aug 4;26(15):7519. doi: 10.3390/ijms26157519 (PMC12347334; doi:10.3390/ijms26157519)
Supplement: Supplementary file 1 [file ijms-26-07519-s001.zip › ijms-3764557-supplementary.pdf]

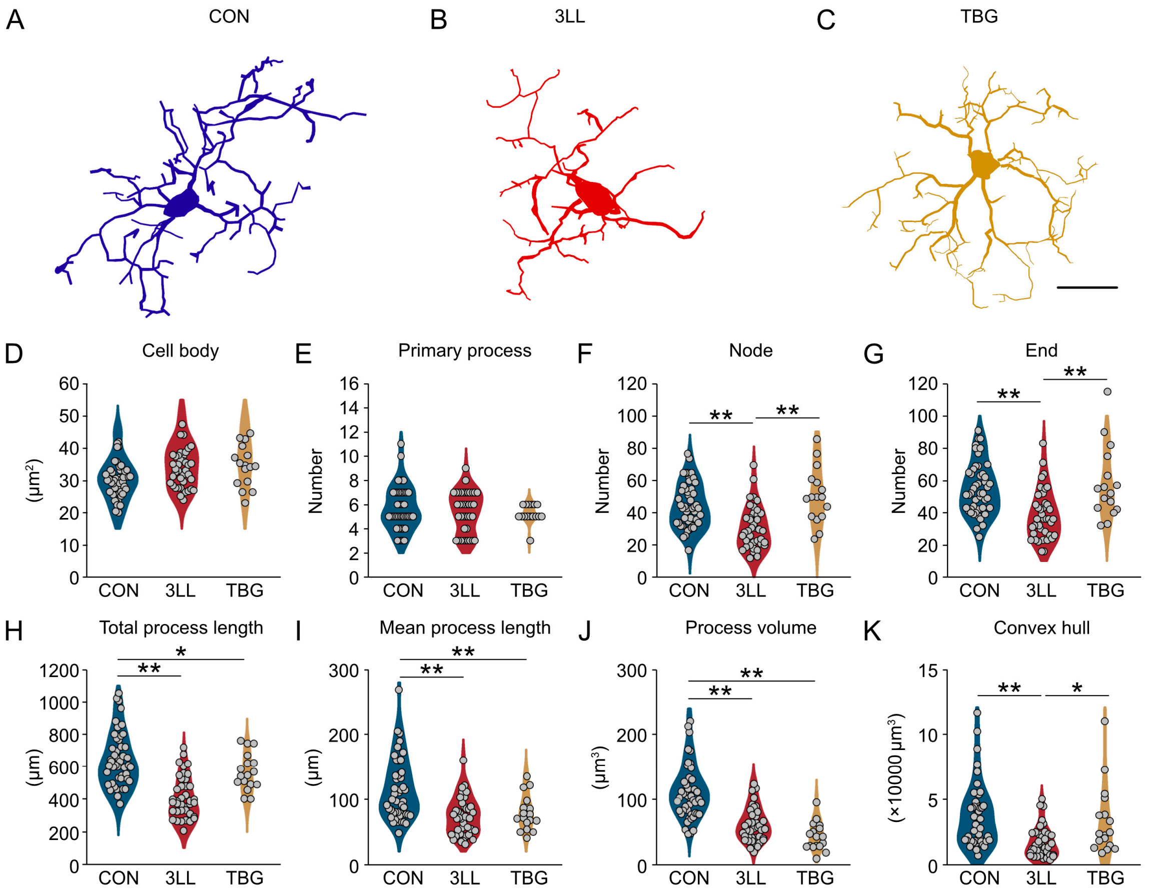

Figure S1. TBG induces morphological remodeling of microglia in the hippocampus of 3LL mice. (**A–C**) Representative computer-assisted tracings of $Iba1^{+}$ microglia from CON (**A**), 3LL (**B**), and TBG (**C**) mice. (**D**) Microglial cell body area (μm²). (**E–G**) Number of primary processes (**E**), process nodes (**F**), and process endpoints (**G**). (**H–J**) Total branch length (μm) (**H**), mean process length (μm) (**I**), and process volume (μm³) (**J**). (**K**) Convex hull volume (×10,000 μm³) formed by microglial processes. Statistical differences were analyzed using one-way ANOVA with Tukey's HSD post-hoc test. Data are presented as mean ± SD (CON, $n = 57$ cells; 3LL, $n = 60$ cells; TBG, $n = 20$ cells). Each grey circle represents an individual cell. Asterisks denote statistical significance: $^{*}p < 0.05$, $^{**}p < 0.01$. Scale bar in (**C**) = 10 μm (applies to **A–C**).

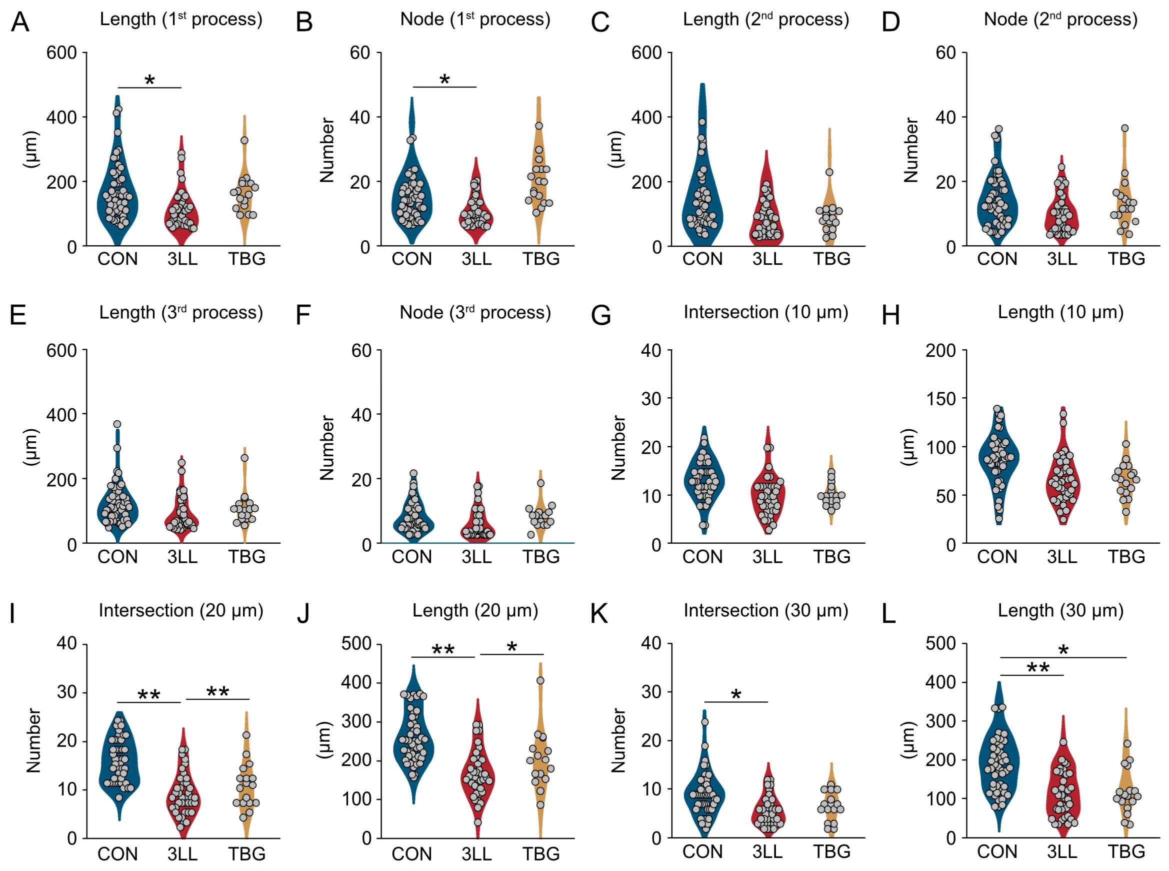

Figure S2. TBG restores microglial process morphology in the hippocampus of 3LL mice. (**A, B**) Length of the first-order process (μm) (**A**) and number of nodes on the first-order process (**B**). (**C, D**) Length of the second-order process (μm) (**C**) and number of nodes on the second-order process (**D**). (**E, F**) Length of the third-order process (μm) (**E**) and number of nodes on the third-order process (**F**). (**G-L**) Summary of Sholl analysis. Number of intersections (**G**) and intersection length (μm) (**H**) with concentric circles at 10 μm radii from the center of the cell body. (**I, J**) Number of intersections (**I**) and intersection length (μm) (**J**) at 20 μm radii. (**K, L**) Number of intersections (**K**) and intersection length (μm) (**L**) at 30 μm radii. Statistical differences were analyzed using one-way ANOVA with Tukey's HSD post-hoc test. Data are presented as mean ± SD (CON, n = 57 cells; 3LL, n = 60 cells; TBG, n = 20 cells). Each grey circle represents an individual cell. Asterisks denote statistical significance: *$p < 0.05$, **$p < 0.01$.

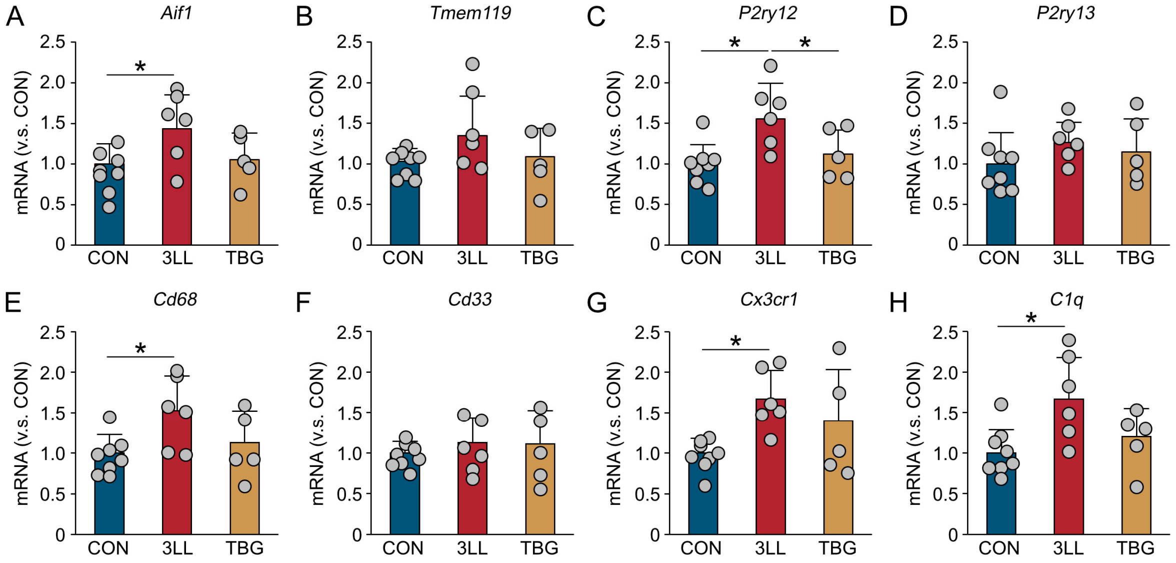

Figure S3. TBG downregulates the expression of genes associated with microglial activation in the hippocampus of 3LL mice. (**A–H**) Relative mRNA expression levels (normalized to CON) of genes related to microglial function in the CA1 region of the hippocampus: *Aif1* (**A**), *Tmem119* (**B**), *P2ry12* (**C**), *P2ry13* (**D**), *Cd68* (**E**), *Cd33* (**F**), *Cx3cr1* (**G**), and *C1q* (**H**). Statistical differences were analyzed using one-way ANOVA with Tukey's HSD post-hoc test. Data are presented as mean ± SD (CON, n = 8 mice; 3LL, n = 6 mice; TBG, n = 5 mice). Each grey circle represents an individual animal. Asterisks denote statistical significance: *p < 0.05.

Table S1. Statistical summary of the Welch's *t*-test (Figs. 1-4).

Figure No.	Variables	*t* value	*p* value
1B	Tumor size (D8)	$t_{19.32} = 0.535$	0.599
1B	Tumor size (D11)	$t_{15.65} = 0.267$	0.793
1B	Tumor size (D14)	$t_{16.07} = 0.988$	0.337
1C	Distance traveled (OFT)	$t_{11.35} = 1.682$	0.120
1D	Center zone (OFT)	$t_{13.67} = 2.914$	0.012
1E	Alternation score (YMT)	$t_{11.67} = 0.443$	0.666
1F	Closed arm (EPM)	$t_{10.67} = 2.251$	0.047
1G	Open arm (EPM)	$t_{12.19} = 2.118$	0.055
1H	Open arm ratio (EPM)	$t_{10.67} = 2.356$	0.043
1I	Distance (FST)	$t_{11.94} = 1.472$	0.167
1J	Immobile time (FST)	$t_{10.16} = 0.411$	0.690
1K	Conditioning trial 1 (FC)	$t_{9.777} = 0.841$	0.420
1K	Conditioning trial 2 (FC)	$t_{6.14} = 0.877$	0.413
1K	Conditioning trial 3 (FC)	$t_{9.99} = 1.403$	0.191
1L	Context A (FC)	$t_{11.05} = 2.319$	0.041
1M	Context B (FC)	$t_{9.03} = 1.962$	0.081
1N	Cue (FC)	$t_{11.72} = 0.920$	0.377
2B	Tryptophan (Blood)	$t_{7.86} = 2.760$	0.025
2C	Kynurenine (Blood)	$t_{7.19} = 3.360$	0.008
2D	Tryptophan (Brain)	$t_{10.31} = 2.474$	0.032
2E	Kynurenine (Brain)	$t_{10.77} = 0.891$	0.393
2F	*Tph2*	$t_{9.36} = 2.335$	0.043
2G	*Ido1*	$t_{6.94} = 1.054$	0.327
2H	*Ido2*	$t_{7.75} = 1.992$	0.083
2I	*Tdo2*	$t_{8.11}= 0.082$	0.937
3A	*Htr2a*	$t_{9.99} = 2.330$	0.042
3B	*Htr3*	$t_{7.77}= 0.855$	0.418
3C	*Htr4*	$t_{10.11} = 4.500$	0.001
3D	*Il1b*	$t_{6.59} = 2.44$	0.047
3E	*Tnf*	$t_{11.61} = 2.57$	0.025
3F	*Il6*	$t_{10.80} = 2.25$	0.046
3I	Iba1 (so)	$t_{5.28} = 5.830$	0.002
3J	Iba1 (sp)	$t_{6.29} = 4.900$	0.002
3K	Iba1 (sr)	$t_{5.68} = 5.567$	0.002
4G	5-HT	$t_{26.98} = 2.322$	0.028
4H	Iba1	$t_{18.72} = 3.293$	0.004
4I	5-HT/Iba1	$t_{22.76} = 2.684$	0.013

Table S2. Statistical summary of the one-way ANOVA (Figs. 5-7).

Figure No.	Variables	*F* value	*p* value
5C	Distance traveled (OFT)	$F_{2,33} = 1.481$	0.242
5D	Center zone (OFT)	$F_{2,33} = 3.333$	0.049
5E	Alternation score (YMT)	$F_{2,33} = 2.705$	0.082
5F	Closed arm (EPM)	$F_{2,33} = 2.308$	0.117
5G	Open arm (EPM)	$F_{2,33} = 3.309$	0.050
5H	Open arm ratio (EPM)	$F_{2,33} = 3.522$	0.042
5I	Distance (FST)	$F_{2,33} = 1.100$	0.346
5J	Immobile time (FST)	$F_{2,33} = 2.044$	0.147
5K	Conditioning trial 1 (FC)	$F_{2,15} = 1.479$	0.261
5K	Conditioning trial 2 (FC)	$F_{2,15} = 0.276$	0.762
5K	Conditioning trial 3 (FC)	$F_{2,15} = 0.519$	0.606
5L	Context A (FC)	$F_{2,15} = 5.242$	0.017
5M	Context B (FC)	$F_{2,15} = 3.291$	0.067
5N	Cue (FC)	$F_{2,15} = 2.174$	0.147
6A	*Htr2a*	$F_{2,12} = 5.572$	0.019
6B	*Htr3*	$F_{2,12} = 0.727$	0.503
6C	*Htr4*	$F_{2,12} = 1.112$	0.363
6D	*Il1b*	$F_{2,12} = 4.732$	0.031
6E	*Tnf*	$F_{2,12} = 4.732$	0.031
6F	*Il6*	$F_{2,12} = 2.807$	0.100
6J	Iba1 (so)	$F_{2,15} = 6.497$	0.009
6K	Iba1 (sp)	$F_{2,15} = 1.504$	0.253
6L	Iba1 (sr)	$F_{2,15} = 4.494$	0.029
7B	*Il1b*	$F_{2,12} = 31.169$	< 0.001
7C	*Tnf*	$F_{2,12} = 93.798$	< 0.001
7D	*Il6*	$F_{2,12} = 8.667$	0.005
7E	*Aif1*	$F_{2,12} = 67.248$	< 0.001
7F	*P2ry12*	$F_{2,12} = 115.407$	< 0.001

Table S3. Statistical summary of the one-way ANOVA (Figs. S1-S3).

Figure No.	Variables	*F* value	*p* value
S1D	Cell body	$F_{2,98} = 1.579$	0.211
S1E	Primary process	$F_{2,98} = 0.500$	0.608
S1F	Node	$F_{2,98} = 15.584$	< 0.001
S1G	End	$F_{2,98} = 12.548$	< 0.001
S1H	Total process length	$F_{2,98} = 30.826$	< 0.001
S1I	Mean process length	$F_{2,98} = 15.155$	< 0.001
S1J	Process volume	$F_{2,98} = 42.270$	< 0.001
S1K	Convex hull	$F_{2,98} = 10.387$	< 0.001
S2A	Length (1st process)	$F_{2,98} = 7.015$	0.001
S2B	Node (1st process)	$F_{2,98} = 4.744$	0.011
S2C	Length (2nd process)	$F_{2,98} = 2.575$	0.082
S2D	Node (2nd process)	$F_{2,98} = 3.019$	0.054
S2E	Length (3rd process)	$F_{2,98} = 2.230$	0.113
S2F	Node (3rd process)	$F_{2,98} = 2.753$	0.069
S2G	Intersection (10 µm)	$F_{2,98} = 2.933$	0.059
S2H	Length (20 µm)	$F_{2,98} = 2.333$	0.104
S2I	Intersection (20 µm)	$F_{2,98} = 28.332$	< 0.001
S2J	Length (20 µm)	$F_{2,98} = 23.384$	< 0.001
S2K	Intersection (30 µm)	$F_{2,98} = 9.149$	< 0.001
S2L	Length (30 µm)	$F_{2,98} = 18.299$	< 0.001
S3A	*Aif1*	$F_{2,16} = 4.462$	0.029
S3B	*Tmem119*	$F_{2,16} = 1.585$	0.235
S3C	*P2ry12*	$F_{2,16} = 5.169$	0.019
S3D	*P2ry13*	$F_{2,16} = 0.964$	0.403
S3E	*Cd68*	$F_{2,16} = 3.647$	0.049
S3F	*Cd33*	$F_{2,16} = 0.228$	0.080
S3G	*Cx3cr1*	$F_{2,16} = 4.479$	0.029
S3H	*C1q*	$F_{2,16} = 3.733$	0.047
